# Prevalence and associated factors for climatic droplet keratopathy in Kazakhs adults: a cross-sectional study in Tacheng, Xinjiang, China

**DOI:** 10.1186/s12886-021-02065-4

**Published:** 2021-08-30

**Authors:** Zhixiang Hua, Xiaoyan Han, Guoqing Li, Li Lv, Xiaolan He, Laman Gu, Jianfeng Luo, Jin Yang

**Affiliations:** 1 Department of Ophthalmology and the Eye Institute, Eye and Ear, Nose, and Throat Hospital, Fudan University, 83 Fenyang Rd., Shanghai, People’s Republic of China; 2 Key NHC Key Laboratory of Myopia (Fudan University), Laboratory of Myopia, Chinese Academy of Medical Sciences, Shanghai, 200031 China; 3 Shanghai Key Laboratory of Visual Impairment and Restoration, Shanghai, China; 4 Ninth Division Hospital of Xinjiang Production and Construction Corps, Xinjiang, China; 5 Emin County People’s Hospital, Xinjiang, China; 6 Department of Biostatistics, School of Public Health, Fudan University, 130 Dongan Rd., Shanghai, 200032 China; 7 NHC Key Laboratory of Health Technology Assessment (Fudan University), Shanghai, China; 8 Key Laboratory of Public Health Safety of Ministry of Education (Fudan University), Shanghai, China

**Keywords:** Climatic droplet keratopathy, Prevalence, Risk factors

## Abstract

**Background:**

Investigation of the prevalence of climatic droplet keratopathy (CDK) in Tacheng, Xinjiang, China.

**Methods:**

A total of 1030 participants, in their 40s or older, from the Kazakh ethnic group in Tacheng, were randomly sampled by stratification method. Ophthalmic examinations and surveys were carried out on these participants. Factors associated with CDK were analyzed with logistic regression models.

**Results:**

CDK was found in 66 (6.4%; 95% confidence interval [CI]: 4.9–7.9%) Kazakh individuals.

After multiple regression model analysis, it demonstrated that age (< 0.001), exposure time (< 0.001), exposure protection (< 0.001), and vegetable intake (< 0.001) were of correlation with CDK, of which age (OR = 1.21[CI]: 1.16–1.27) and long-term outdoor exposure (OR = 2.42[CI]: 1.26–4.67) were the risk factors, and that vegetable intake (OR = 0.29[CI]: 0.14–0.59) and wearing a hat (OR = 0.24[CI]: 0.10–0.56) were protective factors.

**Conclusions:**

This study has revealed the risk and protective factors of CDK, providing a new insight on related research.

**Supplementary Information:**

The online version contains supplementary material available at 10.1186/s12886-021-02065-4.

## Background

Climatic droplet keratopathy (CDK) is also known as the spheroidal keratopathy, Labrador keratopathy, or Bietti’s keratopathy [1–3]. CDK is characterized by corneal opacity which results from a increment of oil-like deposits on the anterior elastic lamina and anterior stromal layer [4]. Previous studies have indicated that the prevalence of CDK varies widely in different regions and ethnic groups, with rates as low as 2.7% [5] and as high as 100% [6]. Zhang et al. (1991) reported that in Inner Mongolia, CDK mainly occurs in middle-aged and elderly Mongolians with the prevalence being 8.1% in adults over the age of 30 [7]. Currently, high-quality research in the field of CDK is absent.

Generally, CDK is considered to be a degenerative corneal disease associated with chronic corneal damage. The exact pathogenesis of CDK is still not clear, but there are many risk factors for CDK including: exposure to ultraviolet radiation, aging, dietary habits, and occupational hazards. Among them, ultraviolet radiation is the most widely established factor [1, 8]. Studies have reported that the intensity and time of exposure to ultraviolet radiation correlates significantly to the prevalence of CDK [1].

Currently, there are no specific pharmacological treatments for CDK, but superficial keratectomy along with amniotic membrane transplant or corneal transplantation are available for advanced CDK cases [9]. However, the procedure is not able to be practiced in several impoverished regions. The epidemiology and pathogenesis of CDK gain little attention because only a few patients seek medical assistance due to its mild symptoms in the early stage [1].

In our clinical work in Tacheng, CDK can only be found among middle-aged and elderly Kazakhs, especially those who have been farming or herding outdoors for many years. The main focus of this study was to identify the most important factors influencing CDK, thus boosting early detection and intervention. To the best of our knowledge, no study has been conducted to investigate the prevalence of CDK among the Kazakh ethnic group in Xinjiang. This study reports the prevalence of CDK and its associated risk factors in the Kazakh ethnic group in Tacheng, Xinjiang, and reviews existing literature on the prevalence of CDK.

## Methods

### Sampling

**Fig. 1:**
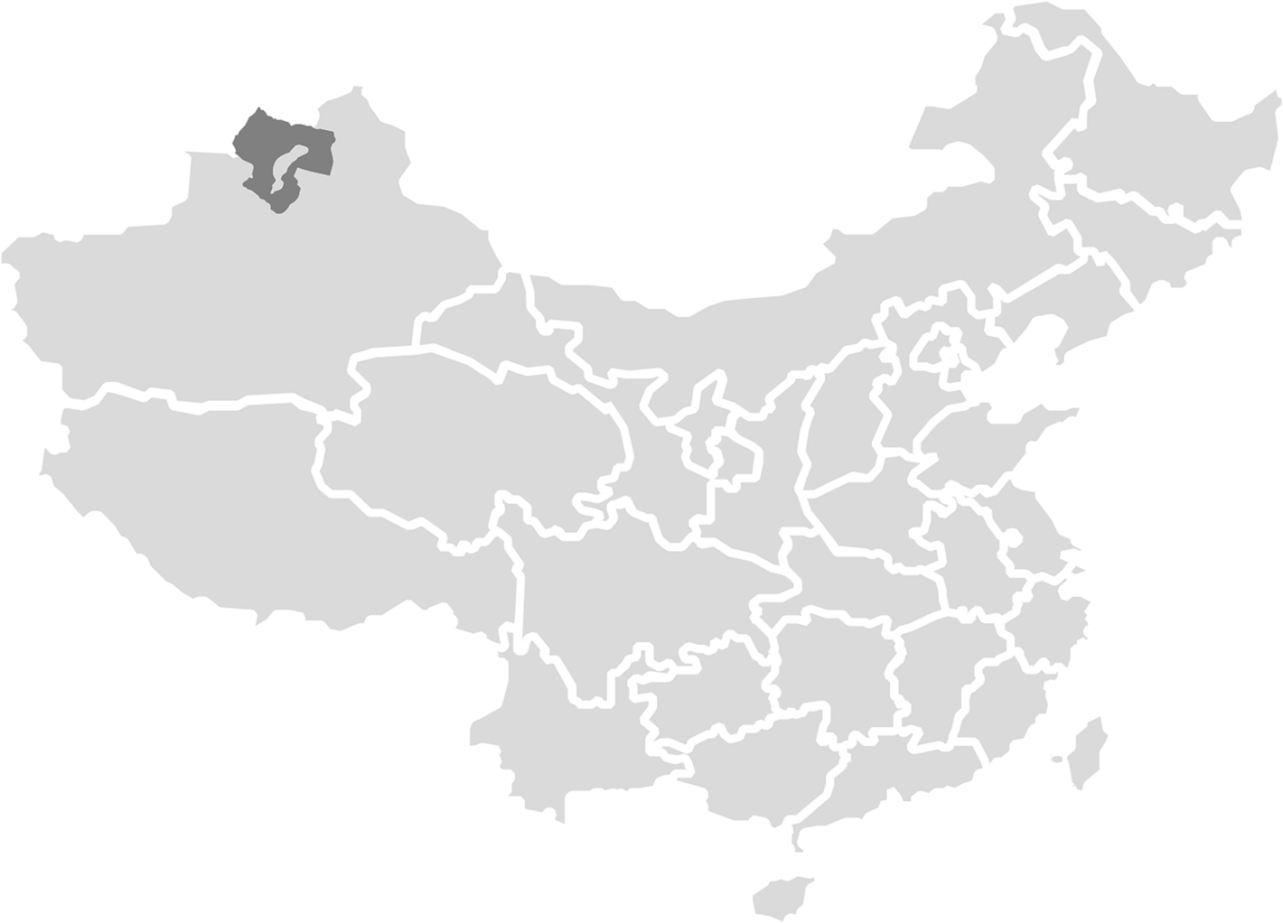
The map of China highlighting site of this work (Tacheng prefecture) (Artificial drawing created by author ZXH)

Tacheng prefecture (longitude 82° 16′- 87° 21′, east; latitude 43 25′- 47 ° 15′, north) (Fig. 1) is in the Northwestern part of Xinjiang Uyghur Autonomous Region, China. It has an area of 98824km^2^, and is divided into two county-level cities (Tacheng and Usu), four counties (Emin County, Shawan County, Toli County, and Yumin County), and an autonomous county (Hoboksar Mongol Autonomous County).

Sample size was calculated with the cross-sectional study formula $$ N=\frac{z_{1-\raisebox{1ex}{$a$}\!\left/ \!\raisebox{-1ex}{$2$}\right.}^2p\left(1-p\right)}{d^2} $$, where Z_1-α/2_ = 1.96. As α = 0.05, p stands for the prevalence of CDK (which was 5.0% in our pretest), and d is admissible error (which is 2%). According to the formula, the theoretical sample size was 502, which includes an extra 10% for subject’s loss.

We have randomly selected 7 different townships in different cities and counties. The final selection was made by assigning each township with a number and use a random number generator from WinPepi to select numbers within the range of the township list. A public hospital was chosen in each township for participants examination.

The study included only Kazakh residents who had lived in the area for more than 5 years and aged 40 years or above. Monocular patients were excluded. In addition, the study also excluded patients with a history of ocular surgery or ocular trauma and other diseases such as mental illness which could potentially affect the evaluation of CDK. The study was based on the principles of the Declaration of Helsinki. Ethical approval was granted by the bioethical committee of the Ninth Division Hospital of Xinjiang Production and Construction Corps.

### Data collection

Data was collected from September 2019 to December 2019 by a team of ophthalmologists and other medical staff from The Ninth Division Hospital of Xinjiang Production and Construction Corps. During the data collection process, each participant underwent a comprehensive interview by trained interviewers.

The questionnaire comprised basic personal information, lifestyle habits, and medical history. Basic personal information included name, age, gender, ethnicity, occupation (farmer, herder, worker, housework, and others), dietary habits (vegetables, fruits, meat, and milk tea), lifestyle (smoking and alcohol consumption), and disease history (e.g., hypertension, hyperlipidemia, diabetes, arthritis, and emphysema). Vegetables refer only to leafy green vegetables. We listed several types of local vegetables in the questionnaire. (see supplementary questionnaire)Agricultural and animal husbandry work were identified as outdoor occupations, while the others were considered as indoor occupations. Height and weight were measured in centimeters and kilograms respectively. Body mass index (BMI) was calculated using the universally recognized formula: weight (kg) / height (m^2^). Systolic and diastolic blood pressures were measured with a digital automatic blood pressure monitor. The average of the three measurements was recorded.

The questionnaire was written in Chinese, and each Kazakhs participant had undergone a comprehensive interview by trained interviewers, who is proficient in Kazakh and Chinese. Before the preliminary experiment, our questionnaire was reviewed by Dr. Luo, an expert on epidemiology and statistics. We have conducted a preliminary experiment and tested our questionnaires at a sheep farm (Township-level administrative unit) in Emin County, Tacheng Prefecture, with 121 participants in total. The results showed that the questionnaire had a Cronbach’s alpha of 0.83 and Kaiser-Meyer-Olkin (KMO) of 0.842, which demonstrates good questionnaire reliability and validity.

The anterior segments of all participants were examined carefully with a slit lamp (YZ5E, Six-six, Suzhou, China), followed by the recording of all uncorrected visual acuity and abnormal performance of the conjunctiva, cornea, lens, etc. The diagnosis of CDK (in either eye) was based on the clinical symptoms and slit-lamp examinations, and the degeneration was divided into three stages (Table 1) [1, 2].

**Table 1:** Diagnostic criteria [1, 2]

Grade	Status	Findings
0	Normal	No abnormal changes under the slit lamp
1	Slightly abnormal	Under the slit lamp, tiny sub-epithelial deposits can only be seen on the nasal or temporal limbus
2	Abnormal	Under the slit lamp, there are more obvious drop-like deposits, involving more than 2/3 of the cornea, and the cornea below the horizontal line of the pupil can be changed in a mist.
3	Significantly abnormal	The sediment is fused into a sheet, covering the cornea in a strip-shaped lateral direction, sometimes it can also be raised above the normal corneal epithelium, and the corneal surface can be seen with amber nodules

### Statistical analysis

All statistical analyses were performed using the SPSS software program (Statistical Package for Social Sciences Inc., Chicago, IL, USA, Version 21.0.0.0). The data was presented as figures using GraphPad Prism 7.0. The prevalence of CDK and the age-adjusted prevalence was calculated with references from the 2010 China Population Census. For independent samples, t-test and chi-square test were used to analyze the demographic characteristics and the grades of CDK. In addition, univariate analysis between the presence of CDK and prevalent factors was performed. The factors showing statistical or clinical significance were included in multiple logistic regression models. A *P* value of < 0.05 was considered to be statistically significant.

## Result

**Fig. 2:**
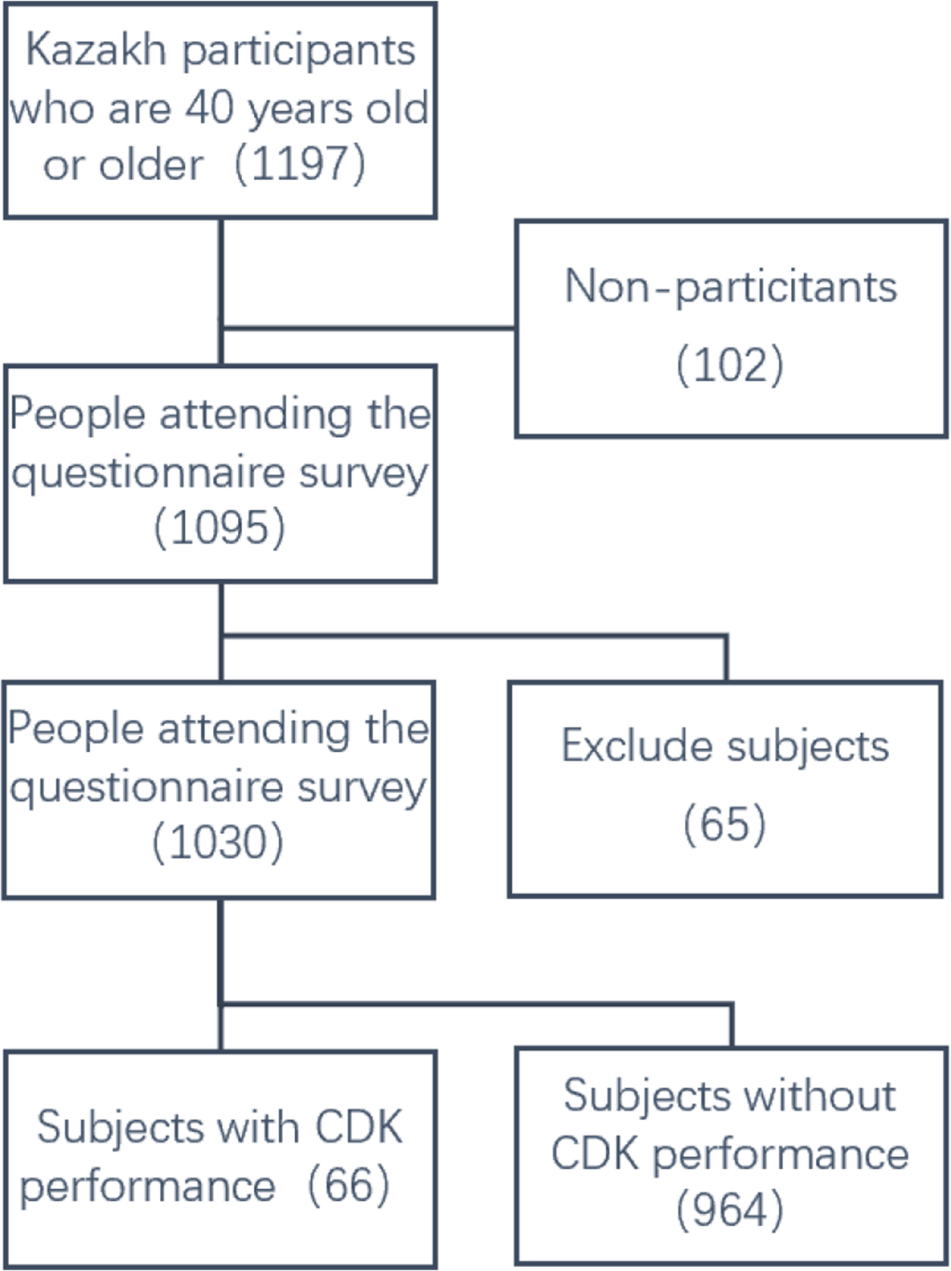
The flow chart of our study

Out of 1197 eligible Kazakh residents, 1095 underwent ophthalmo-logical examinations, with an overall response rate of 91.48%. We finally included 1030 people who were 40 or older (as seen in the flow chart (Fig. 2)). The mean age of the participants in the study was 54.59 ± 9.58 years old with their ages ranging from 40 to 93 years, and 46.9% (*n* = 483) of them were women. CDK was found in 66 (6.4%; 95% confidence interval [CI]: 4.9–7.9%) subjects where 5 (7.6%) of them were unilateral while 61 (92.4%) were bilateral. A comparison of the patient baseline characteristics between CDK group and normal group (non-CDK group) is illustrated in Table 2. And the distribution of CDK in different townships is illustrated in supplementary table. The results indicate that the overall prevalence (P) of CDK among the population was 6.4% (95% CI: 4.9–7.9%), and the prevalence of CDK was higher in men than women (8.9% vs. 4.2%, *p* < 0.05).

**Table 2:** Characteristics of the Kazakh participants (CDK vs. Normal group)

	CDK group	Normal group	*P* value
Number	66	964	
Sex			=0.003
Male	43	440	
Female	23	524	
Age(range)	55–93	40–86	
Age(year), Mean ± SD	69.50 ± 7.12	53.57 ± 8.85	< 0.001
Height(cm), Mean ± SD	165.26 ± 9.35	162.37 ± 9.45	< 0.001
Weight(kg), Mean ± SD	69.47 ± 11.88	71.48 ± 12.40	< 0.001
BMI, Mean ± SD	25.40 ± 3.54	27.15 ± 4.59	< 0.001
Occupation			0.010
Indoor	12	324	
Outdoor	54	640	
Exposure time	2.67 ± 0.69	2.08 ± 0.65	< 0.001
Light(0-4 h/d)	6	160	
Moderate(4-8 h/d)	8	548	
Heavy(> 8 h/d)	50	256	
Exposure protect			< 0.001
Glasses	2	68	
Hat	11	392	
Other	1	28	
None	52	476	
Vegetable intake	1.26 ± 0.51	1.74 ± 0.54	< 0.001
Light(< 300 g/d)	51	300	
Moderate(300-500 g/d)	13	619	
Heavy(> 500 g/d)	2	45	
Vegetable species	3.43 ± 1.62	5.85 ± 2.62	< 0.001
Fruit intake	1.09 ± 0.34	1.22	< 0.001
Light(< 200 g/d)	59	756	
Moderate(200-350 g/d)	4	204	
Heavy(> 350 g/d)	1	4	
Meat intake	2.18 ± 0.90	1.98 ± 0.50	< 0.001
Light(0-120 g/d)	18	128	
Moderate(120-200 g/d)	14	728	
Heavy(> 200 g/d)	32	108	
Smoking			
Number of people	18	356	0.115
Intake, Mean ± SD	0.64 ± 1.00	0.63 ± 1.00	0.989
Alcohol			
Number of people	19	280	0.964
Intake, Mean ± SD	9.88 ± 28.77	22.383 ± 48.08	0.121
Tea milk intake	2.30 ± 0.86	2.31 ± 0.91	0.864
None(0bowl/d)	3	56	
Light(1-3bowl/d)	8	128	
Moderate(4-6bowl/d)	21	240	
Heavy(>6bowl/d)	34	540	
Hypertension, n%	43	508	0.050
Diabetes, n%	3	88	0.204
Hyperlipidemia	9	232	0.053
Emphysema	1	68	0.082
Arthritis	34	388	0.072
Cholecystitis	0	40	0.091
Pterygium	24 (36.4%)	244 (25.3%)	0.048
Cataract	41 (62.1%)	340 (35.3%)	< 0.001

**Fig. 3:**
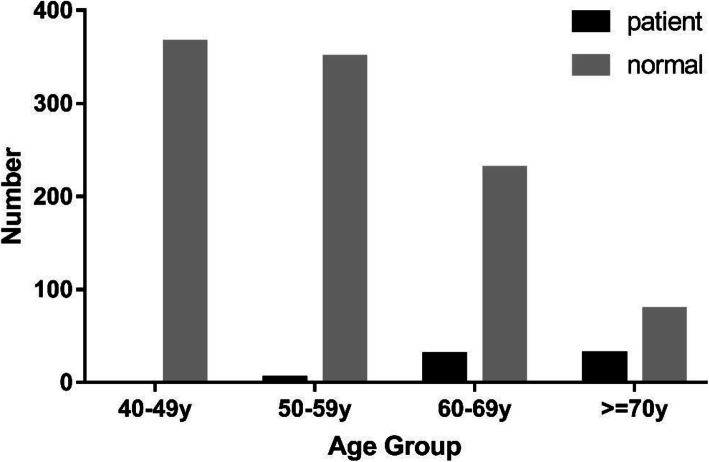
Distribution of residents among the different age group

**Fig. 4:**
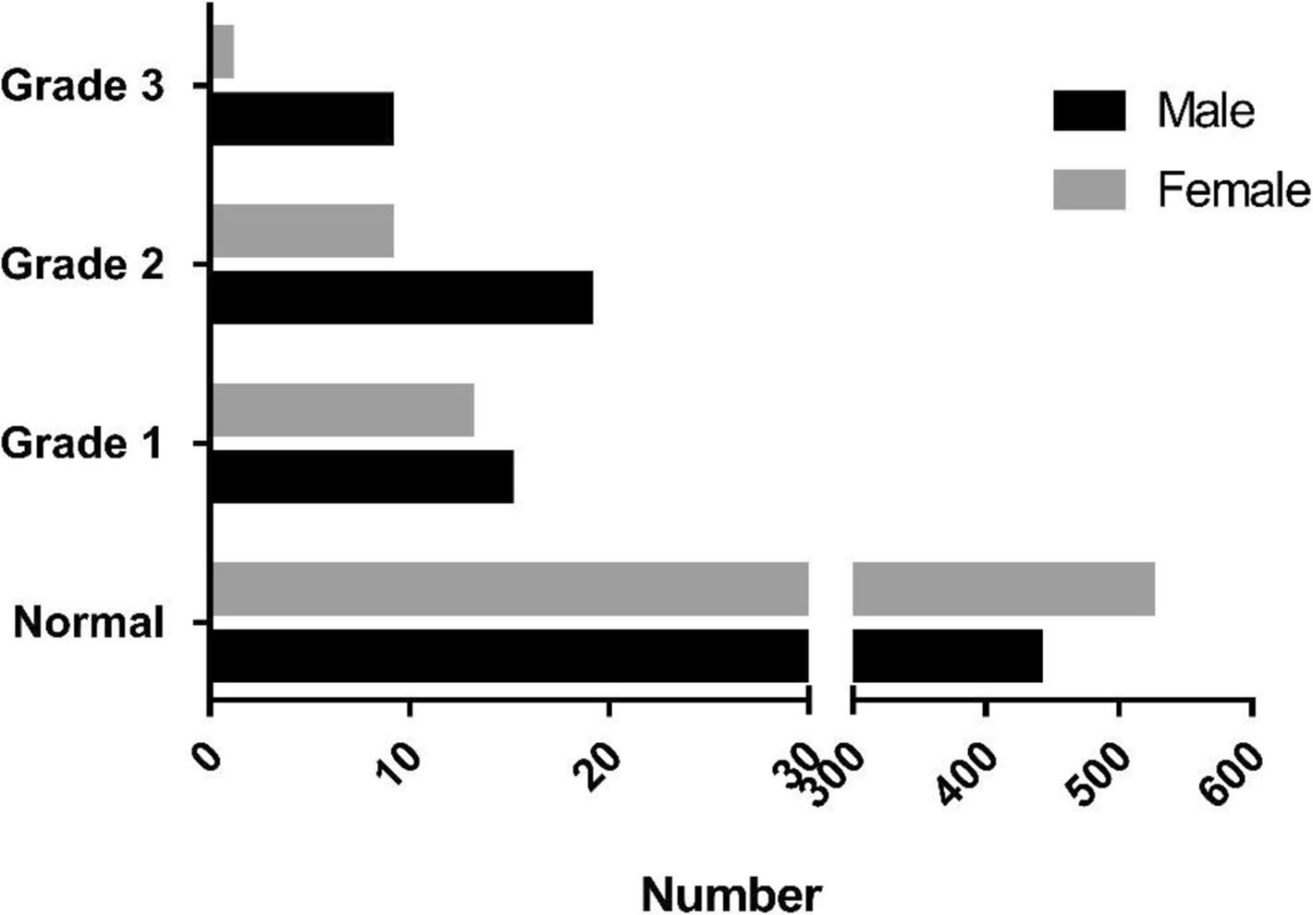
Distribution of residents in different grade and gender

Fourteen out of all sixty-six patients diagnosed with CDK in this study are in different stages of the disease respectively in the two eyes, nine patients had a binocular disease, and five patients had monocular disease with the other eye being normal. However, patients with CDK in one eye were also included in the patient group to harmonize the data, and the disease stage was determined using the more severe eye. Among all CDK patients, 28 patients (42.4%) were classified as Grade 1, 28 patients (42.4%) were in Grade 2, and 10 patients (15.2%) were in Grade 3. The distribution of the three grades of CDK with regards to age and gender is shown in Figs. 3 and 4. The mean logMAR for the CDK patients’ uncorrected visual acuity is 0.87 ± 1.06. The mean logMAR in grade 1, grade2 and grade3 is 0.69 ± 0.90, 1.02 ± 1.22, 0.91 ± 0.97.

**Fig. 5:**
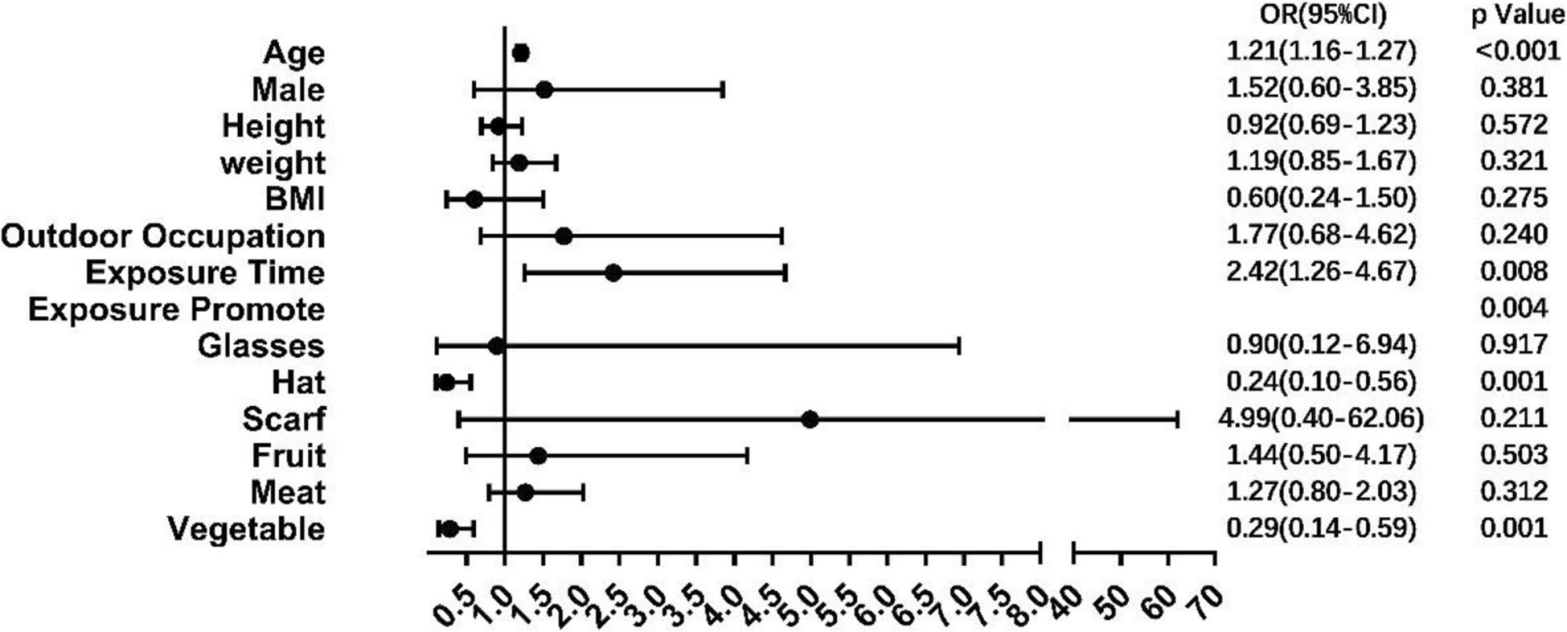
Multivariate analysis of factors associated with CDK

Logistic regression model was used to evaluate the relationship between the occurrence of CDK and the significant factors in Table 2 (*P* < 0.05). The results are presented in Fig. 5. The results found that age and exposure time were the risk factors for CDK whereas exposure protection (especially hat-wearing) and vegetable intake were the protective factors.

## Discussion

This work conducted the first cross-sectional study on the prevalence of CDK in Xinjiang with its results indicating that the prevalence rate among the subjects was 6.4%. We speculated that the prevalence in this study may be slightly lower than clinical expectations. Based on existing literature, the prevalence rate of CDK depends on the environment, climate, and medical condition. Results obtained from studies done among the areas with reported cases of CDK indicate that the prevalence of CDK varies widely in different regions, ethnic groups, and education background. Table 3 provides an overview of the previous studies done on the prevalence of CDK. However, only studies done after 1980 are included since the definition and grading standards of CDK in earlier research were unclear.

**Table 3:** A summary of other worldwide studies concerning CDK

Study	Year	Sample size	Country	Addition	Age	prevalence
1 [10]	1980	82	Australia	aborigine	aged 33 and above	43.9%
2^a^ [6]	1981	2500 1100 42,000	Labrador^a^	Inuit Indian Caucasians	aged 40 and above	64–95% 77–100% (11–100%)
3 [11]	1981	350	Australia		Of all age	14.9%
4 [12]	1984	189 127 659 810	Japan	(Kyoto) (Jordan) (Greenland) (Denmark)	Of all age	30.7% 39.4% 12.3% 4.3%
5 [13]	1984	646	Labrador		unclear	19.0%
6^b^ [14]	1988	3241	Chad		unclear	0.2–1.6%
7 [15]	1989	838	Maryland	watermen	aged 20 and above	19.3%
8 [16]	1989	1519	Transkei		Of all age	11.7%
9 [17]	1991	1117 1329	Republic of Djibouti	urban rural	aged 40 and above	0.5% 2.8%
10 [7]	1991	1949	China	Inner Mongolia	aged 30 and above	8.1%
11 [18]	1994	4312	Mongolia		aged 40 and above	26.5%
12 [5]	1995	225	Rwanda		of all age	2.7%
13 [19]	2006	577	Argentine	Patagonia	of all age	7.6%
14 [20]	2014	1054	Ethiopia	Southwest Ethiopia	Of all age	4.4%
15 [21]	2015	125	Argentina		aged 20 and above	20.0%
16 [22]	2017	5012	India	Western Rajasthan	aged 40 and above	10.7%

^a^This article does not provide raw data and describes it separately by latitude and gender. This result only represents the prevalence range of different races

^b^We fail to find the full text of this article, the information is based on the abstract on PubMed

Johnson (1981) reported that the peak prevalence of CDK occurred between a latitude of 55 and 56 degrees and the UVR in Labrador reached a peak almost exactly on the same latitude [6]. This study was conducted in Tacheng prefecture (43° 25′- 47° 15′, north) which has a lower latitude than Labrador, therefore, the prevalence of CDK was significantly lower than that in Labrador. Intriguingly, the prevalence of CDK in this study was consistent with the prevalence in Inner Mongolia [7]. However, it was significantly lower than that of Mongolia. It is worth noting that Xinjiang, Mongolia, and Inner Mongolia border each other and are on roughly similar latitudes. This indicated that there were some other factors associated with the prevalence of CDK in addition to latitude and UVR.

In this study, the occurrence of CDK was shown to be associated with age, because with the prevalence increasing with age. This finding is consistent with results from all studies listed in Table 3. Based on the positive correlation between CDK prevalence and age, we believe that the prevalence of CDK in Tacheng will increase due to the aging population. As above, the development of CDK has been known to be associated with overexposure to UVR [1]. Chronic exposure to UVR causes actinic keratosis or keratopathies which primarily affects the epithelium and the anterior stroma. The association between CDK and older age can be attributed to the cumulative exposure to UVR and ocular physiologic changes like dryness in people with increased age. This explanation was reinforced by the finding in this study that wearing hats for sun protection was a protective factor against CDK.

The impact of gender on the prevalence of CDK is still controversial. Most studies have shown that the prevalence of CDK is higher in men than in women with the exception being in some earlier studies [23, 24]. The studies attributed the result to the fact that men usually have longer outdoor exposure time than women. Despite there being a difference in the prevalence of CDK among Kazakh men and women (8.9% vs. 4.2%, *p* < 0.05) in this study, it did not constitute a protective or risk factor when further regression analysis was done (*p* > 0.05). We attributed this result to the fact that the Kazakh people in the Tacheng prefecture make a living by grazing. Therefore, men and women are engaged in similar outdoor farming and animal husbandry activities thereby resulting in no significant difference in exposure time between men and women.

Results obtained from this study indicated that there was a relationship between the dietary habits and CDK prevalence. Up to now, most Kazakhs still maintain the traditional nomadic pastoral lifestyle. They prefer eating meat over vegetables and fruits and have retained the habit of drinking a lot of traditional milk tea. Their dietary habits are significantly different from other ethnic groups. Therefore, we simply summarized their eating habits as a compound diet with high fat and low-vegetable content.

Studies have shown that a high-fat diet can cause tear film dysfunction, damaging the patient’s ocular surface, and induces dry eye symptoms [25, 26]. Additionally, several studies have reported that a high-fat diet results in high oxidative stress in the body [27–29], which increases the consumption of antioxidants and enzymes thereby indirectly perfecting conditions for CDK development. Therefore, we concluded that a high-fat diet might be a risk factor for CDK even though in this study meat intake was not a significant risk factor. We speculated that this could be ascribed to the fact that Kazakhs have a high meat intake. A high-fat diet has already resulted in high BMI levels in the sample population (BMI > 24). In addition, a long-term study conducted in Xinjiang indicated that Xinjiang Kazakhs were a high-risk group for metabolic syndrome and cardiovascular diseases [30]. This further confirmed the negative effects of the above diet structure on the Kazakhs.

The role of low-vegetable diet on the occurrence of CDK is prominent when compared with the high-fat diet. Results obtained from this study confirmed that the insufficient variety and quantity of vegetable intake was a risk factor for CDK. Since humans cannot synthesize vitamin C (VC) autonomously, insufficient daily vegetable intake causes a lack of VC in the body. A study in Argentina indicated that CDK patients there had abnormal dietary habits similar to the dietary habits of Kazakhs. In addition, the patients’ serum VC levels were significantly lower than the controls’, which was consistent with results obtained in this study [21]. Vitamin C has both antioxidant and metabolic functions [31, 32], and it also helps in the formation of the collagen structure. Studies have confirmed that VC plays an important role in the cornea’s defense system to counteract the damaging effects of UVR [33, 34]. Therefore, it is evident that the lack of VC increases the risk of CDK. However, further research should be done on the role of dietary habits and nutrients on the occurrence of CDK. It is possible to prevent or delay the development of CDK by changing the existing dietary habits of the patients or supplementing their nutritional deficiencies. Exploration of dietary habits also provides a new direction for the study of the pathogenesis of CDK.

The virtue of this work is that it included the in-depth analysis of life-style and dietary habits. However, this work also had some limitations. First, self-reports by patients may be limited by the accuracy of their recall and different perceptions of disease. Second, the number of patients was relatively small and only a single ethnic population from a single center was evaluated.

## Conclusion

This study investigated the prevalence and associated factors of CDK among Kazakhs in Tacheng, Xinjiang, China. Obtained results indicated that the overall prevalence of CDK was 6.4% and the exposure time and age were risk factors associated with CDK occurrence. In addition, increasing the variety and quantity of vegetable intake and wearing a hat were protective factors for CDK. Based on this study, health education should be emphasized for Kazakh farmers and herdsmen should be advised to change their current dietary structure, increase vegetable intake, and take protective measures such as wearing a hat or sunglasses during outdoor work.

## Supplementary Information

**Additional file 1.** Ethics review.

**Additional file 2.** Questionnaire.

**Additional file 3.** Table: Distribution of CDK in different townships.

## Data Availability

The datasets used and analyzed in the current study are available from the corresponding author upon reasonable request.

## Abbreviations

CDK: Climatic droplet keratopathy
BMI: Body mass index
VC: Vitamin C
UVR: Ultraviolet radiation
P: Prevalence
CI: Confidence interval
