# Supplementary material for: Prevalence and associated factors for climatic droplet keratopathy in Kazakhs adults: a cross-sectional study in Tacheng, Xinjiang, China
Source: BMC Ophthalmol. 2021 Aug 30;21:316. doi: 10.1186/s12886-021-02065-4 (PMC8404251; doi:10.1186/s12886-021-02065-4)
Supplement: Supplementary file 1 — Additional file 1. Ethics review. [file 12886_2021_2065_MOESM1_ESM.docx]

**新疆塔城地区气候性滴状角膜变性的流行病学调查**

您好，为了能够及时完成本次检查的反馈和后续的回访，我们需要采集您的个人信息，感谢您的配合。

1. 基本情况

（一）受调者姓名： 联系电话

（二）家庭住址 抽样点编号

（三）性别： （1） 男 （2）女

（四）年龄: 岁 民族 ： 族

（五）身高： cm 体重： kg 血压： / Bp 血糖 mmol/L

（六）婚姻状况 （1）已婚 （2）未婚

（七）职业：

二、工作方式

（一）工作方式 ： （1）室内工作 年 (办公室，工人，家务，其他)(请打勾)

（2）户外工作 年 (放牧，务农，其他) (请打勾)

（二）日间户外暴露时间：（1） 0-4h /d （2）4-8h/d （3）8h以上/d

（三）日间户外暴露保护：（1） 眼镜 （2）帽子 （3）其他 （4）无

三、饮食习惯(以周为单位估算日均摄入)（200--350g/的水果摄入约为一个苹果或者橙子）

（一）每日水果摄入（苹果 橘子 葡萄 香蕉 瓜）

（1）不足 <200g/d （2）适量200--350g/d （3）过量 >350g/d

（二）日常蔬菜种类调查 （若有则打勾）

土豆 芹菜 菠菜 青菜 青椒 西红柿 洋葱 胡萝卜 大白菜 西兰花

每日绿色蔬菜摄入（如包括土豆在内的上述蔬菜，）

1. 不足 <300g/d （2）适量300--500g/d （3）过量 >500g/d

（三）每日肉类摄入（牛肉 羊肉 鸡肉 猪肉）

（1）不足 <120g/d （2）适量120--200g/d （3）过量 >200g/d

四、其他习惯

（一）有无抽烟习惯（1支每天并持续1年） （1）有 （2）无（跳过此题）

日抽烟量 （1）轻度 <10支/d （2）中度10-20支/d （3）重度 >20支/d

（二）有无饮酒习惯（1次每周并持续1年） （1）有 （2）无（跳过此题）

饮酒习惯： 啤酒 白酒 （请将受访者日常饮用的酒类打勾）

啤酒 小瓶（330ml 酒精量10.5g） 瓶/日 大瓶（550ml 17.6g） 瓶/日

白酒 1两（ 50ml 酒精量19.2g） 两/日

（三）有无饮奶茶习惯（1次每日并持续1年） （1）有 （2）无（跳过此题）

日奶茶摄（以300ml为一碗（杯）计量）

（1）轻度：2-3碗/日 （2）中度：4-6碗/日 （3）重度 ：7碗及以上/日

五、健康情况（若有相关疾病，简要描述病程及病情，如双侧膝关节炎5年）

（一）高血压病史 （1）无 （2）有，

（二）高血糖病史 （1）无 （2）有，

（三）高血脂病史 （1）无 （2）有，

（四）肺气肿病史 （1）无 （2）有，

（五）膝关节炎病史 （1）无 （2）有，

（六）其他病史 （肝炎结核等传染病史，心脑血管病史，重大外伤或相关病史）

六、专科检查

|  | 右眼情况 | 左眼情况 | 评级说明（请将对应的数字填入左侧表格中） |
| --- | --- | --- | --- |
| 裸眼视力 |  |  |  |
| 睑裂宽度 |  |  |  |
| 泪液分泌测试 |  |  |  |
| 泪膜破裂时间 |  |  |  |
| CDK分级 |  |  | 0.正常，1级，2级，3级 |
| 翼状胬肉 |  |  | 0.正常 1.结膜期 2.角膜前期  3.进展期 |
| 白内障 |  |  | 0.正常 1. 皮质性 2.核性  3.后囊下 4.其他类型 |
| 皮质性白内障 |  |  | 0.正常 1.初发期 2.未成熟期 3.成熟期 4.过熟期 |
| 其他眼科疾病 |  | | |

调查日期：

调查人签名：
