# Supplementary material for: Prevalence and associated factors for climatic droplet keratopathy in Kazakhs adults: a cross-sectional study in Tacheng, Xinjiang, China
Source: BMC Ophthalmol. 2021 Aug 30;21:316. doi: 10.1186/s12886-021-02065-4 (PMC8404251; doi:10.1186/s12886-021-02065-4)
Supplement: Supplementary file 2 — Additional file 2. Questionnaire. [file 12886_2021_2065_MOESM2_ESM.docx]

**Epidemiology Survey of Climatic Droplet Keratopathy in TaCheng, XinJiang**

Greetings, for the benefit of time in completing this survey and later return visit, we will need to collect your personal info, thank you for your cooperation.

1. Basic Status
2. Name of surveyed： Contact Number
3. Family address Sample Number
4. Gender (1) Male (2) Female
5. Age: Ethnic:
6. Height: cm Weight: kg Blood Pressure: / Bp

Blood Sugar: mmol/L

1. Marital Status (1) Married (2) Single
2. Occupation:

2. Working Conditions

1. Working conditions:

(1) Indoors for Years (office, worker, domestic chore, others) (Tick Please)

(2) Outdoors for Years (grazing, farming, others) (Tick Please)

b) Day-time out door exposure time (1) 0-4h /d (2) 4-8h/d (3) More than 8h/d

c) Day-time out door exposure protection: (1) Eye Wear (2) Hat (3) Others

(4) None

3. Diet Habit(Estimate daily consumption based on weekly consumptions) (200-350g/of fruit intake is about an apple or orange)

a) Daily fruit intake （apples, oranges, grapes, bananas and melons）

(1) Insufficient <200g/d (2) Moderate 200--350g/d (3) Overdose >350g/d

b) Daily vegetable intake variety survey (Tick if present)

Potato Celery Spinach Chinese cabbage Green pepper

Tomatoes Onions Carrots Cabbage Broccoli

Daily vegetable intake quantity survey (vegetables above, including potatoes)

(1) Insufficient <300g/d (2) Moderate 300--500g/d (3) Overdose >500g/d

c) Daily Meat consumption（beef, mutton, chicken and pork）

(1) Insufficient <120g/d (2) Moderate 120--200g/d (3) Overdose >200g/d

4. Other Habits

a) Smoking (1 pcs per day, lasting for the past year) (1) Yes (2) No (Please Skip)

Daily Smoking status (1)Light <10pcs/d (2)Moderate 10-20pcs/d (3)Heavy >20 pcs/d

b) Drinking (1 time per week, lasting for the past year) (1) Yes (2) No (Please Skip)

Drinking Habit: Beer Spirit (Tick the ones he/she usually drinks)

Beer Bottle (330ml 10.5g Alcohol) pcs/day

Jar (550ml 17.6g Alcohol) pcs/day

Spirit 1 Liang (50ml 19.2g Alcohol) Liang/day

c) Milk Tea (1 time per day, lasting for the past year) (1) Yes (2) No (Please Skip)

Daily Milk Tea intake (Measuring in the unit of bowls/cups as in 300ml per bowl/cup)

(1) Light: 2-3 bowls/day (2) Moderate: 4-6 bowls/day

(3) Heavy: More than 7 bowls/day

5. Health Conditions (If related disease is present, please elaborate the condition and the course of the disease, e.g. 2 years of bilateral gonarthitis.)

a) High Blood Pressure (1)No (2)Yes,

b) High Blood Sugar (1)No (2)Yes,

c) Hyperlipidemia (1)No (2)Yes,

d) Pueumonectasis (1)No (2)Yes,

e) Gonarthitis (1)No (2)Yes,

f) Other Conditions (Hepatitis, tuberculosis or other contagious disease, Cardio/Cerebrovascular conditions, Major Trauma or other related conditions.)

6. Specialist examinations

|  | OD | OS | Relating notes (Fill the numbers in the blocks ) |
| --- | --- | --- | --- |
| UCVA |  |  | —————— |
| Palpebral aperture |  |  | —————— |
| Schirmer test |  |  | —————— |
| TBUT |  |  | —————— |
| CDK |  |  | 0. Normal, 1. degree,  2. degree, 3. degree |
| Pterygium |  |  | 0. Normal, 1. Conjunction period , 2. Frontal cornea period, 3. Developing period |
| Cataract |  |  | 0. Normal, 1. Cortical, 2. Nuclear, 3. Under posterior capsule, 4.Others |
| Cortical cataract |  |  | 0. Normal, 1. Incipient stage, 2.Pre-muature, 3.Muature, 4.Over-muature |
| Other Ophthalmology Conditions |  | | |

Survey Date:

Surveyor Sign:
