# Supplementary material for: Prevalence and associated factors for climatic droplet keratopathy in Kazakhs adults: a cross-sectional study in Tacheng, Xinjiang, China
Source: BMC Ophthalmol. 2021 Aug 30;21:316. doi: 10.1186/s12886-021-02065-4 (PMC8404251; doi:10.1186/s12886-021-02065-4)
Supplement: Supplementary file 3 — Additional file 3. Table: Distribution of CDK in different townships. [file 12886_2021_2065_MOESM3_ESM.docx]

| **Supplementary Table: Distribution of CDK in different townships** | | | | | | | |
| --- | --- | --- | --- | --- | --- | --- | --- |
| TOWNSHIP  Name | CHAHETE | WUXUETE | TUOLI | SHANGHU | KAERYEMULE | YEMENLE | GUERTU |
| CDK | 7(0.68%) | 9(0.87%) | 11(10.68%) | 8(0.77%) | 14(1.36%) | 9(0.87%) | 8(0.77%) |
| NORMAL | 113(10.97%) | 150(14.56%) | 146(14.17%) | 130(12.62%) | 164(15.92%) | 131(12.72%) | 130(12.62%) |
| TOTAL | 120(11.65%) | 159(15.44%) | 157(15.24%) | 138(13.40%) | 178(17.28%) | 140(13.60%) | 138(13.40%) |
